# Analysis of short-term temporal variations of ^222^Rn, other naturally occurring radionuclides, stable elements and environmental parameters in groundwater and surface drinking water in Norway

**DOI:** 10.3389/fpubh.2025.1620899

**Published:** 2025-07-09

**Authors:** Aleksander Sverdrup Aarsand, Jelena Mrdakovic Popic, Hans-Christian Teien

**Affiliations:** ^1^Faculty of Environmental Sciences and Natural Resource Management, Norwegian University of Life Sciences, Ås, Norway; ^2^Norwegian Radiation and Nuclear Safety Authority, Østerås, Norway

**Keywords:** radon, naturally occurring radionuclides, groundwater, drinking water quality, temporal variation

## Abstract

**Introduction:**

Exposure to radon-222 (^222^Rn) is a common problem in areas with high uranium-238 (^238^U) content in the subsurface. The gas may enter dwellings through cracks and gaps in the foundation, or groundwater collected for household use. It is well documented that ^222^Rn poses a health risk, especially in high concentrations. In water, the gas often co-occurs with other naturally occurring radionuclides (NOR), such as radium-226 (^226^Ra) and polonium-210 (^210^Po). These may, in combination with chemically toxic elements, negatively affect water quality and consequently human health.

**Materials and methods:**

To investigate ^222^Rn content in drinking water and changes over time, water quality in six sampling points in western Norway were monitored over a period of 17 months. The majority of NORs, stable elements and general water parameters were found to be within accepted limits for drinking water quality in Norway. However, one of the sampling points, a drilled granite well, displayed high activity concentrations of ^222^Rn (up to 1,225 Bq/L), ^210^Pb (up to 41.7 mBq/L) and ^210^Po (up to 312 mBq/L). Water from other sampling points displayed low pH (5.8–6.5), which could affect mobility and bioavailability of toxic elements.

**Discussion:**

The magnitude of variation of ^222^Rn activity concentration was generally reflected in other parameters, such as Ca and ^238^U, but statistically significant correlation (*p* < 0.05) could only be found in three sampling points. Several water parameters, such as Ca, electrical conductivity, ^222^Rn and ^238^U displayed statistically significant correlation (*p* < 0.05) with temperature and precipitation, suggesting a seasonal dependence. Therefore, the variability was attributed to mineral weathering, recharge through rocks and regolith with different NOR-content, and dilution by rapid recharge. The findings of this study show that activity concentrations of ^222^Rn in different types of water sources is affected by recharge patterns, which should be considered for when assessing drinking water quality.

## 1 Introduction

Radon (^222^Rn) is part of the natural uranium (^238^U) decay chain and is therefore present in the environment where primordial ^238^U is found, for example in rocks, such as granites, pegmatites and black shales (1, 2). Radon is a noble gas and has a much higher mobility in the environment compared to solid radionuclides. It has a relatively short half-life of 3.82 days, and the emanation rate from the mineral phase is proportional to concentration of radium (^226^Ra) and grain size of the solid phase (3). Radon may travel through permeable rocks and regolith before entering dwellings through cracks and gaps in the foundation. Radon is well-known health risk, and according to World Health Organisation (4) the second cause of the lung cancer worldwide, just after tobacco smoking. However, during exposure, ^222^Rn itself is mostly exhaled, while a significant amount of the received dose comes from its short-lived daughters, alpha-emitters that easily attached to aerosol particles (5). These may deposit on bronchial epithelium and are known to increase the risk of lung cancer even at moderate concentrations, and especially in combination with smoking (6, 7). According to European legislation, i.e., Council Directive 2013/59 (EU BSS) the indoor airborne activity concentration should be below 300 Bq/L (8). However, several areas in Norway, Sweden, Finland and Denmark are prone to high radon concentrations due to drift geology and ^238^U-content in bedrock, and ^222^Rn activity concentrations upwards of 50,000 Bq/m^3^ have been observed (9, 10).

Subterranean ^222^Rn may also enter aquifers where groundwater is collected for domestic use. In groundwaters, concentrations upwards of several tens of thousands Bq/L has been recorded across Finland, Sweden and Norway (11–13). In these studies, ^222^Rn also showed a geological dependence, usually being highest in consolidated rock wells of ^238^U-bearing minerals. In addition, the activity concentrations in ground water tends to be higher than in surface water as ^222^Rn emanates intro the atmosphere. While the ingestion of waterborne ^222^Rn is believed to only be a small contributor to dose, emanation and subsequent inhalation of the gas is the more important exposure route (14). Several studies have attempted to identify a correlation between increase in stomach, bladder or kidney cancers and higher ^222^Rn activity concentrations implying higher received doses (15–17). However, thus far no association has been found for ^222^Rn concentrations exceeding 300 Bq/L. In Norway, limit values for drinking water quality is described in the Norwegian Drinking Water Regulations, and Regulations on Certain Contaminants in Foodstuffs, which is based on several European regulations (18, 19). Limit values for radionuclides in drinking water are based on Council Directive 2013/51/Euratom (20), which defines a numerical value for ^222^Rn in drinking water of 100 Bq/L, and a minimum requirement to a number of yearly measurements depending on the scale of the supply network. For all other radionuclides, excluding ^222^Rn, tritium, and potassium-40, the directive defines a maximum indicative dose (ID) of 0.1 mSv/year. Thus, the maximum admissible activity concentration of any given radionuclide is dependent on the cumulative dose from other radionuclides. Due to its chemical toxicity, the WHO recommend a limit of 30 μg/L for uranium (21). This limit is not reflected in Norwegian regulations.

Due to its volatility, the transport and accumulation of ^222^Rn in soil-gas and groundwater is typically controlled by physical processes, such as soil-permeability, water table dynamics and climate (3). The general assumption for indoor airborne ^222^Rn is that activity concentration tends to be higher during the cold season, although this is not always the case (10, 22). The explanation for this is geogenic properties and temperature, leading to differences in gas flow (23, 24). In Norway, indoor airborne measurements are conducted during the colder part of the year (October to April) and a correction factor is applied to estimate the yearly average (10). Subterranean ^222^Rn gas may also dissolve in groundwater, both in cracks and pores in rocks and in regolith (3). In the aquatic phase, ^222^Rn content will depend on the rate of solvation and the rate of degassing. The solvation of ^222^Rn in water is governed by water temperature, salinity, organic content, as well as residence time for ground water (25–27). Degassing takes place any time the partial pressure of ^222^Rn is higher in the aquatic phase than the air phase. Bubbling, heating, or simply storing the water are efficient methods for outgassing ^222^Rn. A few studies have investigated temporal variations in waterborne ^222^Rn. In Arnea, Greece, one of the boreholes supplying water to the village displayed some fluctuation over a 5-year monitoring period, with measured activity concentrations in the range 659–1,100 Bq/L and 377–680 Bq/L in the highest activity wells (28). However, these fluctuations were deemed insignificant form a radiation protection perspective. Erlandsson, Jakobsson (29) found that waterborne ^222^Rn content in a 72 m deep well varied from day to day in the range 235–358 Bq/L, with no additional long-term variation over a period of 3 years. De Francesco, Tommasone (30) found seasonal variation in shallow groundwater in southern Italy. In the three monitoring wells, ^222^Rn activity was at its lowest during summer, and increased up to two orders of magnitude in the autumn-spring period. This was attributed to groundwater discharge driven by rainfall events.

The chemical composition of surface waters is subject to rapid changes due to input from different natural and anthropogenic sources. Groundwater is filtered through sediments and is somewhat protected from outside contamination. As the groundwater matures, water quality starts to reflect composition of sediments and aquifer material it comes into contact with. Groundwaters often have altered pH, and elevated concentrations of electrolytes compared to its surface counterpart, limited by the solubility of the mineral phase (31). Composition of subterranean waters may change throughout the year due to variable recharge from precipitation and meltwater (32, 33). While concentrations of major ions, i.e., sodium (Na), magnesium (Mg), potassium (K) and calcium (Ca), are typically associated with aquifer withering, rare earth elements (REEs) are typically associated with particle adsorption and are mobilized during initial recharge by slightly acidic rainfall (34–38). Thus, REE patterns are highly conserved over long groundwater flow paths and can be used to determine mixing and dilution (39, 40).

Several monitoring campaigns have been launched in Scandinavia to evaluate naturally occurring radionuclides in groundwater and surface drinking water (41–44). However, no similar mapping has been done in Norway in recent times. It is well known that water quality is controlled by source type as well as geological factors, which varies between countries. As a result average ion concentrations has been observed to vary greatly (13). Thus, it is assumed that the concentrations and behaviors of radionuclides in Norwegian groundwaters is different to what has been observed in Sweden and Finland.

*We hypothesize that* ^222^Rn in water is controlled by some of the same hydrogeological processes as other elements occurring in groundwater. Water samples have been collected from a ^222^Rn affected area of the Caledonian nappes in Norway to obtain data on ^222^Rn, ^238^U, ^226^Ra, ^210^Pb, ^210^Po, as well as 52 stable elements and anions, temperature, pH, electrical conductivity (EC) and total organic carbon (TOC), and analyze their possible correlations and behaviors. The main aim of the present study has been to investigate seasonal variation in radon and naturally occurring radionuclides (NOR) alongside other water parameters and improve the relatively limited knowledge on this issue, important to apply proper radiation protection measures where needed.

## 2 Materials and methods

### 2.1 Study area and sampling

**Figure 1:**
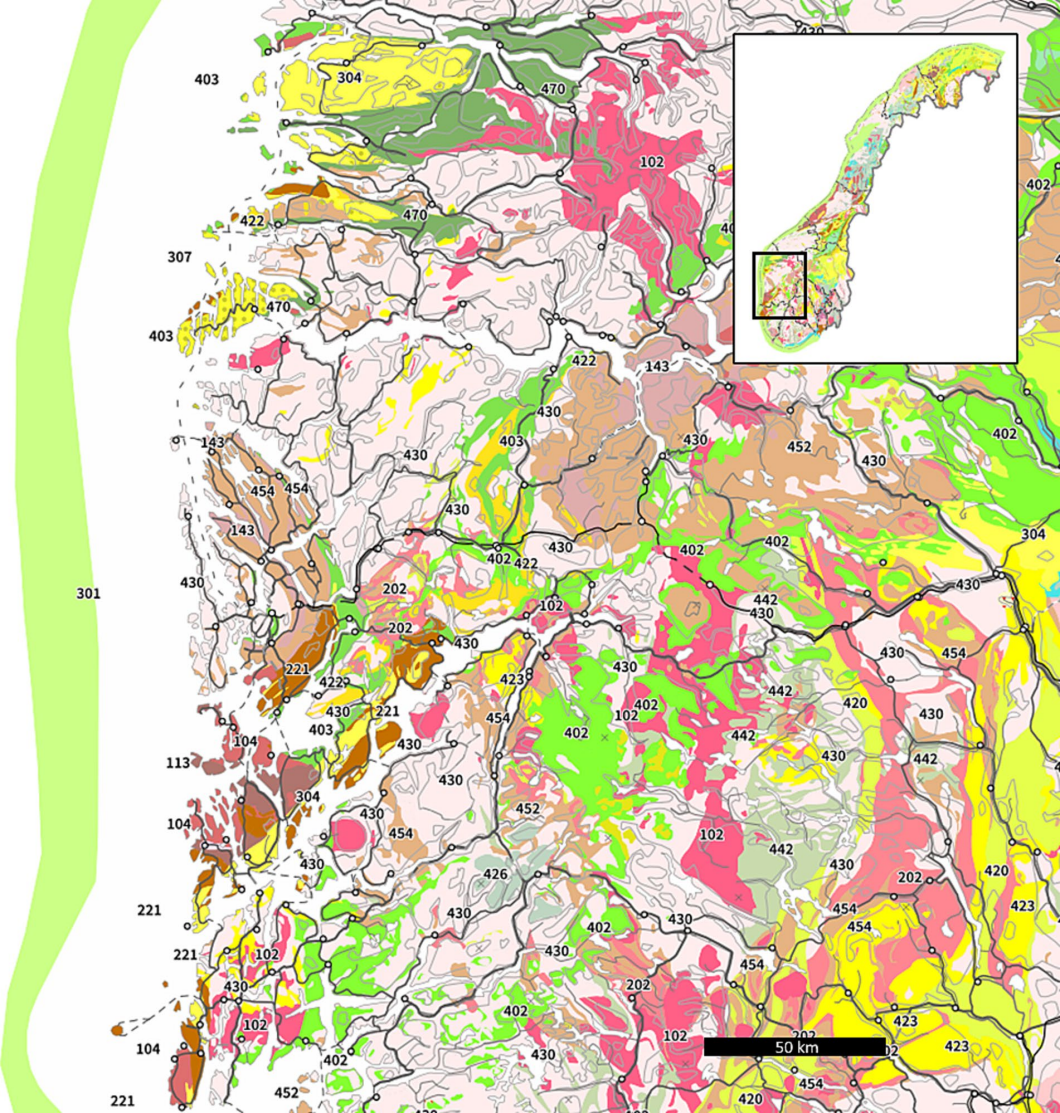
Bedrock map of western Norway including the Caledonian nappes. Granites are denoted with code 102 and rhyolite code 202. Adapted from (45).

The study area and sampling points were identified using online geological maps, which contains data under the Norwegian license for public data (NLOD), made available by the Geological Survey of Norway (NGU). These maps show major bedrock types (see Figure 1) and the estimated indoor radon potential across Norway (45, 46). However, due to the low density of registered wells in some areas, and to avoid identification of participants in this study, the exact sampling locations are not marked.

The study area itself is a sparsely populated area, part of the Caledonian nappes in western Norway. High indoor ^222^Rn has been recorded previously and five anonymous volunteers plus one waterwork agreed to participate in water quality monitoring of the current study. The sampling points are located in the same general area, within 10 km of each other in steep terrain. The catchment area likely extends to the top of the hillside (800–1,000 meters above sea level) and there is no industrial activity and limited human activity upstream of the collection points. According to the bedrock maps of NGU (Figure 1), geology of the area consists of granite and rhyolite, which are chemically similar rock types known for containing ^238^U (47, 48). The cover material of the study area is dominated by glaciofluvial deposits and avalanche material (49). Information about the water wells was collected from the national groundwater database GRANADA (50). In order to investigate how water quality in different water sources vary over time, six sampling points were selected:

1. A drilled granite rock well located about 150 m above sea level. According to the bore log, the well is approximately 100 m deep and had a flow rate of 600 L/h before pressure blasting.
2. A drilled rhyolite rock well located about 110 m above sea level. According to the bore log, this well is approximately 100 m deep and had a flow rate of 500 L/h before pressure blasting. The log also states that the last 30 m of the well was drilled due to a problem with leakages.
3. An unconsolidated well located about 100 m above sea level (hereinafter referred to as sampling point A of the unconsolidated well). This well is located 30 m away from a river in an area dominated by moraine and fluvial deposits on rhyolite bedrock. Thus, the well is recharged by intrusion from the river and precipitation in the surrounding area. The well itself is a little over 1 m deep.
4. Following the outlet used for the previous sampling point, the water is UV-treated and aerated in an open reservoir before being distributed to a handful of households. Samples were collected from a household supplied by the unconsolidated well, located approximately 600 m away from the source (hereinafter referred to as sampling point B of the unconsolidated well).
5. A household drawing water from a spring source located in steep terrain between 250 and 300 m above sea level, and about 900 m from the sea. The regolith in the area around the spring is composed mainly of avalanche material.
6. A household drawing water from a nearby stream. This water runs from at least 900 m above sea level and is collected at approximately 175 m.

Following the initial sampling conducted in June of 2023, the owners of sampling points 1 through 5 agreed to participate in monitoring of water quality over time. Before the sampling in September of the same year, sampling point 6 the granite well, was identified and was also included in monitoring over time. Sampling frequency was largely dictated by planning and coordination with the volunteers. In order to assess the evolution of drinking water quality between winter and summer, samples were collected monthly from January through July of 2024. The last sampling, conducted in November of 2024, was performed shortly after a heavy precipitation event in order to investigate the effects of large volumes of rainwater. The final sample number ended up being 8 for the granite well and stream source, and 9 for other sampling points.

### 2.2 Sample preparation and measurement

Meteorological data from the study area was collected from a publicly database available through the Norwegian Centre For Climate Services (51). Values for total precipitation and average air temperature from the month prior to each sampling were used to assess seasonal dependency of water quality. Measurements of pH, electric conductivity (EC) and water temperature were performed on site using a WTW Multi 3,401 combined pH and EC meter with a Sentix 41 and Tetracon 325 electrode. In order to determine total organic carbon (TOC) untreated samples were collected in 10 mL tubes. The samples were analyzed using a Shimadzu TOC-analyzer.

Stable elements, ^226^Ra, ^232^Th and ^238^U were measured by ICP-MS. Before ICP-MS analysis, samples were acidified to 5% v/v HNO_3_ The analysis of ^226^Ra was done on Perkin Elmer NexION 5,000, and ^238^U, ^232^Th, as well as the content of 52 major-and trace elements in water samples was determined using the Agilent 8,900 QQQ ICP-MS. Anion chromatography was performed on 1.5 mL of untreated sample. Quantification of fluoride (F^−^), chloride (Cl^−^), sulfate (SO_4_^2−^), and nitrate (NO_3_^−^) concentrations were done on a Dionex ICS-6000 HPIC.

The activity concentration of polonium-210 (^210^Po) was determined by *α*-spectrometry from 10 L water samples. Polonium was concentrated using iron hydroxide co-precipitation and spontaneous deposition on nickel disks following a method adapted from Chen, Aarkrog (52) and Skipperud, Jørgensen (53). Another isotope of polonium (^209^Po) was used as yield monitor. Analyses were done on Ortec Model 7,401 spectrometers. The samples were then left for approximately 6 months, allowing the ingrowth of ^210^Po before the process was repeated. This allowed for the determination of lead-210 (^210^Pb) using Equation 1 (54):


(1) $$A_{\mathrm{Pb}}=A_{\mathrm{Po}}\frac{T_{\mathrm{Pb}}-T_{\mathrm{Po}}}{T_{\mathrm{Pb}}}{\left(e^{\frac{\ln0.5*t}{T_{\mathrm{Pb}}}}-e^{\frac{\ln0.5*t}{T_{\mathrm{Po}}}}\right)}^{-1}$$

where A_Pb_, is the estimated activity concentration of ^210^Pb, A_Po_ is the measured activity concentration of ^210^Po, T_Pb_ and T_Po_ are the half-lives of ^210^Pb and ^210^Po respectively, and t is the time elapsed between the first and second separation.

**Figure 2:**
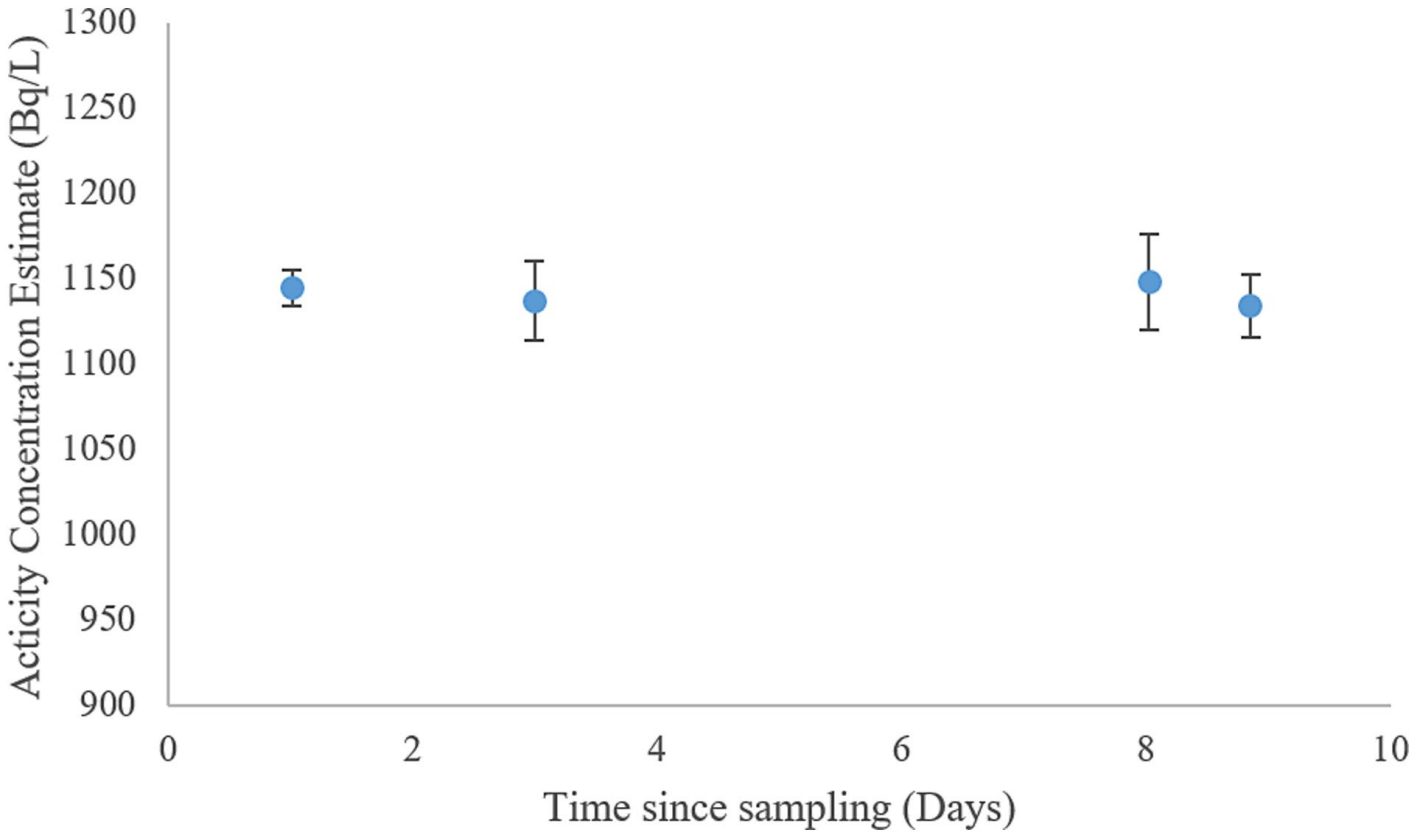
Activity concentration estimate of ^222^Rn samples at different times of storage (*n* = 3).

Due to the volatility of ^222^Rn-gas, loss of analyte during and after sampling is a common problem (55) Samples used for determining ^222^Rn activity concentrations were collected by a method adapted from Strand and Lind (56). Ensuring minimal air contact, water samples were taken into scintillation vials prefilled with Maxilight water immiscible scintillation cocktail. The samples were sealed using hinge tape and aluminum (Al) lined caps and stored in the dark at room temperature for no more than 3 days. Sample stability was tested by storing three samples taken from the granite well in room temperature for just under 9 days and estimating activity concentration of ^222^Rn (Figure 2). No meaningful loss of analyte could be identified in the relevant time frame due to storage alone. Analysis of ^222^Rn was done using a Hidex 600SLe liquid scintillation counter. The samples were counted at 17°C for 30 min. The estimated counting efficiency was 2.688 as ^222^Rn, ^218^Po and ^214^Po are all counted.

The radiological risk from NOR in each sampling point was assessed by calculating ID according to Equation 2 using effective dose coefficients found in (57) and assuming a consumption rate of 730 L/y (20):


(2) E=a∗e∗730L/y

where E is the ID in Sv/y, a is the activity concentration of a certain NOR in water, e is the effective dose coefficient for adults.

### 2.3 Statistical analysis

To investigate covariance between different parameters over time, correlation coefficients were calculated in R (v.4.4.1). Due to challenges in planning and coordination of sample collection, the final dataset is limited. To avoid making inaccurate assumptions about data distribution, all statistical analyses were done using non-parametric statistical tests ranked data. Spearman’s rho does not require data to be normally distributed, and was used for measuring the monotonic association between pairs of variables. Since analyses were performed continuously throughout the monitoring period, most of the studied variables had multiple overlapping reporting limits. For radiometric analyses decision thresholds (DT) and detection limits (DL) are calculated according to De Felice, Jerome (58). For mass-based measurement techniques, limits of detection (LOD) and quantifications (LOQ) are calculated as three and ten times the standard deviation of method blanks, respectively. To enable statistical analysis on variables with one or more censored observations, Kendall’s rank test on U-scores was used instead (59). Correlation was considered significant when *p* < 0.05.

## 3 Results and discussion

### 3.1 General water quality

The vast majority of the 65 parameters investigated in this study area were within the norm values according to Norwegian drinking water regulations (18, 19). The main constituents of these waters are the alkaline and alkaline-earth metals, as well as Cl^−^, NO_3_^−^, SO_4_^2−^ (Table 1). The concentrations of these vary between the sampling points as well as over time. The measured concentration of F-in the granite well was relatively high compared to other sampling points, but still no values above the limit of 1.5 mg/L were measured. The concentration of SO_4_^2−^ was also higher in this sampling point than in any other measured, although it remains unclear whether this is due to a difference in kinetic factors, or due to the different mineral compositions of the aquifers. Water from both the granite and rhyolite rock well displayed high Ca concentration at times when compared to the other sampling points. While this may cause issues in terms of usage, it is not associated with negative health effects (60). Water from the rhyolite displayed a larger range in observed EC and concentration of major constituents, although the variations in pH and temperature were comparable to that found in the granite well. In the rhyolite well, the concentration of arsenic (As) varied between 5.6 and 7.5 μg/L, but was never observed above the 10 μg/L limit. Concentrations of Al up to 110 μg/L was also observed, which is higher than most other sample points, but below the drinking water limit of 200 μg/L. Although water quality from the two rock wells were similar at times, it seems the rhyolite well is, to a greater degree, affected by outside influence.

**Table 1:** General water parameters and major constituents of water from each sampling point (Median [min – max]).

Parameter	Unit	Granite	Rhyolite	Unconsolidated A	Unconsolidated B	Spring	Stream
pH		7.4 [7.0–7.9]	7.2 [6.8–7.6]	6.3 [6.0–6.7]	6.6 [6.2–7.0]	7.0 [6.5–7.1]	6.3 [5.8–6.6]
EC	μS/cm	169.7 [167–175.7]	116.4 [95.8–161]	27.9 [24–37.3]	30.5 [22–37.4]	47.9 [43.0–51.0]	16.5 [14.0–25.0]
Temperature	°C	12.9 [8.9–16.0]	10.8 [8.7–15.7]	7.3 [5.4–11.1]	10.7 [4.5–16]	11.4 [7.8–14.2]	10.05 [5.9–14.9]
TOC	mg/L	0.68 [0.42–0.68]	1.7 [1.1–7.2]	0.89 [0.61–1.5]	1.1 [0.67–7.3]	0.85 [0.6–2]	3.5 [2.7–8.5]
F^−^	mg/L	1.0 [0.66–1.4]	0.17 [0.12–0.25]	<0.10	<0.10	<0.10	<0.10
Cl^−^	mg/L	3.9 [2.6–4.0]	3.4 [3.0–3.9]	2.0 [1.2–2.5]	2.0 [1.2–2.7]	2 [1.5–3.1]	2.1 [1.4–3.2]
NO3^−^	mg/L	0.46 [<0.10–0.50]	0.96 [0.42–1.3]	2.1 [0.73–3.3]	2.0 [0.8–4.0]	1.7 [0.71–2.9]	0.48 [0.23–1.3]
SO4^2−^	mg/L	20 [13–24]	3.8 [3.3–11.02]	1.5 [1.3–2.3]	1.6 [1.3–2.4]	3.7 [3.1–4.6]	0.89 [0.56–1.9]
Na	mg/L	7.9 [7.5–10]	3.6 [2.9–4.0]	1.6 [1.3–1.9]	1.6 [1.3–1.9]	2.1 [1.8–2.7]	1.5 [1.4–1.7]
Mg	mg/L	1.0 [0.90–1.1]	1.6 [1.2–1.9]	0.53 [0.38–0.68]	0.50 [0.37–0.65]	0.64 [0.55–0.73]	0.26 [0.22–0.35]
K	mg/L	2.9 [2.5–3.1]	2.4 [2.2–2.7]	0.87 [0.68–1.0]	0.91 [0.67–1.1]	1.5 [1.4–1.6]	0.35 [0.18–0.43]
Ca	mg/L	22 [21–23]	19 [14–24]	2.1 [1.6–2.7]	2.4 [1.7–2.9]	4.6 [3.9–5.2]	0.87 [0.81–1.0]

Between the two sample points in the unconsolidated well, there is little change in the parameters listed in Table 1. However, certain elements appear to differ slightly in concentration: apart from the last measurement, the concentration of iron (Fe) varied between 3.8 and 6.4 μg/L in sampling point A, and varied between 2.0 and 3.0 μg/L in sampling point B. Similarly, manganese (Mn) varied between 0.27 and 0.92 μg/L in sampling point A, and 0.19 and 0.38 μg/L in sampling point B. Both metals are redox sensitive and are likely to precipitate during storage in contact with air, a mechanism that is used for removal of the metals (31, 61). This could also simply be caused by random variation and the distance between the sampling points, as in the last samples collected from these points, concentration of Fe was 10 and 22 μg/L, and the concentration of Mn was 0.72 and 1.2 μg/L in sampling point A and B, respectively. This coincides with the lowest measured pH value of 6.0 and may be caused by the extreme meteorological precipitation leading up to the sampling day. In comparison, the spring source is located on similar bedrock, in steeper terrain, but lacking the recharge from nearby surface waters. It would, therefore, be expected that the water from the spring is slightly more mature than water from the unconsolidated well, something that is reflected in a higher EC and higher concentrations of SO_4_^2−^, Na, K and *Ca.* Water from the stream source had the lowest measured EC, pH, and concentrations of SO_4_^2−^, Mg, K and *Ca.* This is also where the highest TOC was found, an indication of high biological input. Compared to the other sources, the low ion concentration indicates limited interaction between this water and the mineral phase, and the water is exposed to atmospheric and biological input comparable to the rhyolite well and sampling point B in the unconsolidated well. Also similar to the rhyolite well, high concentrations of Al up to 170 μg/L were measured in the stream water. The two sampling points are located less than 1 kilometer apart and may have similar mineral phases even though the waters undergo different processes before being collected.

Differences in water quality between the sampling points are likely explained by the different source types. The lowest observed pH and EC were found in the unconsolidated well and the stream. Water from the surface and near-surface is composed in a high degree of rainwater, i.e., slightly acidic and low in electrolytes (31). Meanwhile, groundwater from the spring, and especially the rock wells have higher pH and EC due to higher dissolution of minerals during contact time. This is comparable to a study by Banks, Frengstad (11), where the median pH in a high number of groundwater samples from different types of crystalline aquifers was found to be 8.07, and slightly lower pH-values in quaternary groundwater and surface waters. The difference in pH was attributed to carbonate mineral dissolution in the crystalline wells. Consequently, mineral dissolution is associated with an increased electrolyte concentration, and thus, higher EC (62, 63). The range of measured EC in the rhyolite well was much greater than in the granite well, indicating less stable conditions over time.

### 3.2 Activity concentration of ^222^Rn and NOR in water and estimation of associated radiation exposure doses

The highest observed concentration of ^238^U was 13 μg/L, the equivalent of 0.16 Bq/L, and was found in the granite well. This is higher than the 3.26 μg/L median found in groundwater drinking water in Sweden, but well below the recommended limit of 30 μg/L, based on its chemical toxicity (21, 43). According to Knutsson (44) the highest concentrations of ^238^U and ^222^Rn measured in Scandinavian groundwaters are 12,400 μg/L and 77,500 Bq/L respectively, both found in Finland.

Measured concentrations of ^232^Th varied between <LOD and 0.11 μg/L. The highest concentration is equivalent to 0.45 mBq/L and was recorded in water from the rhyolite well. Thorium has very low solubility under most conditions and the highest reported concentrations in Norwegian drinking waters are in the range 3.1 and 4.76 μg/L (38, 64). Concentrations of ^226^Ra were never observed above the LOQ of 73 mBq/L in any of the investigated sampling points, which is below the maximum observed activity concentrations in groundwater from Sweden and Finland, which are 2.08 Bq/L and 7.5 Bq/L, respectively (41, 43). Activity concentration of ^210^Pb in the granite well varied between 7.19 and 41.7 mBq/L over time. This is comparable to that found in a study of Finnish groundwaters, where out of 288 samples the median activity concentration of ^210^Pb was 14 mBq/L and the maximum 540 mBq/L (42). In the Finnish ground water study, activity concentrations up to 2.0 Bq/L of ^210^Po where found, while the median value was 9 mBq/L. Meanwhile in the present study, observed activity concentrations of ^210^Po varied by two orders of magnitude, between 2.37 mBq/L and 312 mBq/L.

The radiological risk of naturally occurring radionuclides apart from ^222^Rn was evaluated by calculating the ID in each sampling point (Table 2). A conservative estimate was made using the highest measured activity concentration of each NOR during the sampling period, dose conversion factors were obtained from ICRP publication 119, and assuming a consumption rate of 730 L of water per year (20, 57).

**Table 2:** Measured activity concentrations of ^222^Rn (Median [Minimum-Maximum]) and the yearly dose (mSv/y) from the highest concentration of other measured NORs at each sample point.

Parameter	Unit	Granite	Rhyolite	Unconsolidated A	Unconsolidated B	Spring	Stream
^222^Rn	Bq/L	1,102 [997.4–1,225]	58.8 [31.98–85.4]	13.98 [8.26–16.29]	5.35 [4.11–6.10]	9.54 [8.45–24.79]	0.31[<0.23–0.51]
^238^U	mSv/y	4.0⨯10^−3^	2.2⨯10^−3^	1.4⨯10^−5^	1.5⨯10^−5^	3.6⨯10^−5^	1.0⨯10^−3^
^232^Th	mSv/y	<2.5⨯10^−7^	6.7⨯10^−5^	1.6⨯10^−6^	1.4⨯10^−6^	2.5⨯10^−6^	3.2⨯10^−5^
^226^Ra	mSv/y	<0.015	<6.7⨯10^−3^	<6.7⨯10^−3^	<0.022	<6.7⨯10^−3^	<0.015
^210^Pb	mSv/y	0.02	na	na	na	na	na
^210^Po	mSv/y	0.27	8.9⨯10^−3^	2.1⨯10^−3^	7.9⨯10^−3^	na	na
ID	mSv/y	0.31	0.018	8.8⨯10^−3^	0.030	6.7⨯10^−3^	0.016

For censored values, yearly dose was estimated from the relevant reporting limit. A conservative estimate is made for the ID, assuming the highest possible activity from each NOR.

Large variations in measured activity concentration of ^222^Rn were observed across the sampling points. As expected, the highest activity concentrations of ^222^Rn were found in the rock wells, as waters here typically have longer residence time and are enclosed in the aquifer. Although measured activity concentrations in the granite well greatly exceeded the limit of 100 Bq/L as shown in Table 2, values were in line with observations in other studies of similar areas. Mean values for waterborne ^222^Rn in previous studies of Norwegian wells, primarily drilled in rock, have found a mean value of 400 Bq/L, while Finnish private rock wells tend to be slightly higher, with a mean value of 930 Bq/L (41, 65). A decrease in measured activity concentration of ^222^Rn was observed between sampling point A to B in the unconsolidated well. During aeration and storage of the water ^222^Rn is likely removed through emanation (3, 14).

The National Research Council (14) suggested an effective dose coefficient of 3.5*10^−9^ Sv/Bq for ingestion of ^222^Rn by adults. Using this coefficient, the estimated dose from the highest observed activity concentration is 3.1 mSv/y, making ^222^Rn the greatest contributor to ingested dose, although it is not typically included in calculation of ID (20). Otherwise, the greatest contribution to ID was in most cases from ^210^Po, while those of ^238^U and ^232^Th are negligible compared to the 0.1 mSv/y ID limit. Even though the concentration of ^226^Ra and ^210^Pb is below the derived concentration defined by European Council (20), the dose contribution could be significant depending on the concentration of other NORs not included in this study. Komperød, Rudjord (66) estimated that an average dose of 0.054 mSv/y from all types of drinking water to the Norwegian population, where the estimated dose from ingested ^222^Rn alone was 0.049 mSv/y. In the present study of groundwater in a ^222^Rn-prone area in western Norway, estimated doses from NORs other than ^222^Rn were in the range of 6.7⨯10^−3^ to 0.31 mSv/y exceed the national average of 0.05 mSv/y, while ^222^Rn alone was the greatest contribution to ingested dose.

### 3.3 Temporal variation

Over the course of the monitoring period, water parameters in the different sampling points displayed varying degrees of stability (Table **1**). Meteorological data show that from winter to summer the air temperature increases from just below zero to almost 20°C during summer. The monthly precipitation varies from above 150 mm in January to just above 50 mm in June. Leading up to the final sampling in early November, there was a high rainfall event with 116 mm registered in 1 day. In general, precipitation was higher during the cold season and a significant (*p* < 0.05) negative correlation was found between air temperature and precipitation. However, due to the large catchment area, potential frost during winter, and different types of water source, there is not necessarily a direct connection between precipitation and recharge, although precipitation in the area rarely comes as snow.

The water quality in the granite well was relatively stable throughout the monitoring period. The activity concentration of ^210^Po was at its highest during winter and lowest in summer, although activity concentration of ^210^Pb and ^210^Po show very limited correlation with other variables. A statistically significant (*p* < 0.05) positive correlation was observed between precipitation, SO_4_^2−^, Mg, K, REEs, ^222^Rn and ^238^U. There was also a positive correlation between Na, Rb, Sr. and Cs which correlate negatively with the previous group of variables. Additionally, a positive correlation was found between TOC and Mg, K, Cu, Zn Ba, Sm and Er using Kendall’s rank correlation (*p* < 0.05). The lack of coherence between different group 1 and 2 elements may be caused by anion exchange processes or the presence of minerals with different weathering rates (31). A significant (*p* < 0.05) positive correlation was observed between activity concentration of ^222^Rn and precipitation, possibly indicating that the gas is dissolved in recharge water and transported to the aquifer. A similar observation was made in a karst aquifer in Switzerland (67). The study found that ^226^Ra-content was higher in regolith compared to the underlying bedrock. This led to higher activity concentrations of ^222^Rn in pore-waters and thus higher activity concentrations in the aquifer following recharge. This process could explain the observations made in the granite well in the present study, although a better understanding of the role of catchment geology is needed to be certain.

**Figure 3:**
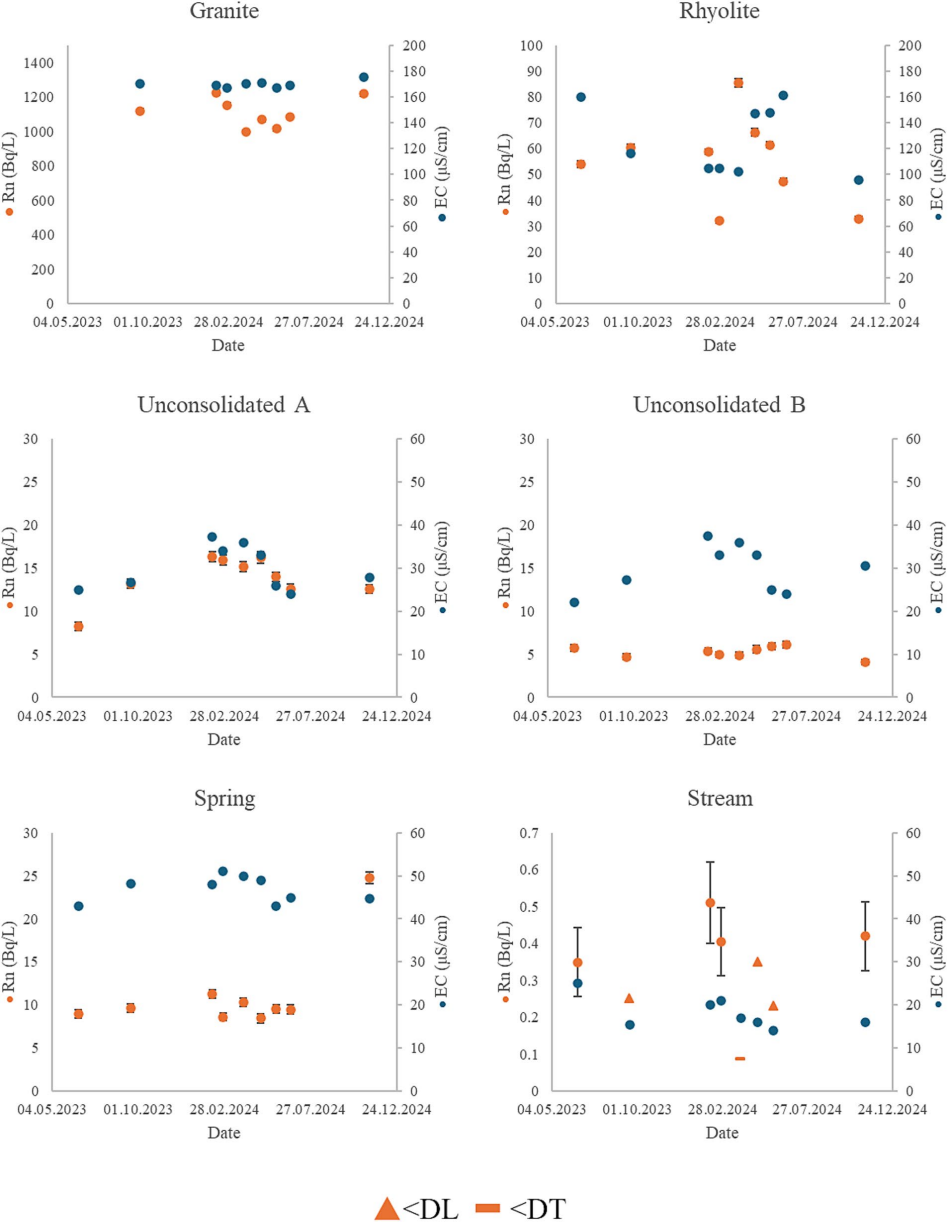
Graphs of ^222^Rn concentration and EC in the six sampling points over the duration of the monitoring period. Values below decision threshold (DT) and detection limit (DL) are marked with the limit value.

Comparably, the conditions in the rhyolite well were less stable as shown in Figure 3. A positive correlation was found between precipitation, TOC, and REEs, as well as between REEs and Al, Mn, Fe, Co and ^232^Th (*p* < 0.05). These metals are typically associated with complexes, colloids or particles, both organic and inorganic (68–70). A significant (*p* < 0.05) negative correlation was found between pH, and REE and ^232^Th, meaning the metals may desorb due to chemical changes. Alternatively, particles may be suspended during influx of rainwater. Meanwhile, there was a negative correlation between precipitation, and EC, Na, Mg, S, K, Ca and ^238^U. Apart from in aquifers composed of highly insoluble minerals, the amount of dissolved minerals in groundwater is typically dependent on residence time alongside surface area, i.e., grain size or fracture geometry (31, 38, 71–73). In periods of high precipitation, the groundwater is possibly diluted by less mature recharge water, leading to lower concentrations of certain metals. High variability in ^222^Rn activity concentration and EC was observed in the rhyolite rock well, as well as the concentrations of several other metals. It has been found that episodic recharge of surface water may lead to rapid changes in water composition (74). During spring this source is likely affected by intrusion from rainwater, as well as meltwater coming from higher parts of the terrain. As ^222^Rn-content might also be affected by such events, it is possible that the lack of correlation with other parameters is due to the time it takes for ^222^Rn to be produced from ^226^Ra-decay.

In sampling point A of the unconsolidated well, temporal variation in ^222^Rn concentration correlated positively with EC, Na, Mg, K and Ca (*p* < 0.05), which may be explained by mixing of surface water and more mature groundwater. A significant negative correlation was observed between ^2122^Rn and air- and water temperature (*p* < 0.05), and during winter there is considerably less water in the nearby river. This leads to less rapid recharge from surface water, meaning more mature water is collected in the well, and thus a higher activity concentration of ^222^Rn and metals associated with mineral dissolution. In sampling point B in the unconsolidated well, seasonal variation in major constituents resembled the patterns observed in sampling point A. The ^222^Rn concentration in sampling point B was, however more stable over time as much of the gas has already emanated during storage, highlighting the usefulness of aeration as a method for ^222^Rn removal (3, 28).

In the spring source there was a negative correlation between air temperature and EC, Na, Mg, K and Ba, indicating a seasonal variation in mixing. It is unknown how this source is recharged, as there are no bodies of water at surface level near the water work. The activity concentration of ^222^Rn remains relatively stable compared to other sampling points, except for the last sampling in November 2024. At this point in time, activity concentration increased two-fold from previous measurements, which is unexpected considering the dilution observed in the unconsolidated well and the rhyolite well. This coincides with the highest measured concentrations of all REEs. Similarly to the granite well, the high activity concentration of ^222^Rn may be caused by recharge water percolating through material with higher ^222^Rn-emanation rate and with longer residence time.

The stream had the lowest measured EC, during the entire monitoring period and comparably low concentrations of most elements. Similarly to the spring and granite well a positive correlation was observed between REEs and air and water temperature, TOC, Al, Ti, V, Cr, As, Zr, Ag, Sb, Pb and U. Although very little ^222^Rn was found in this sampling point, this pattern is similar to that observed in the other sampling points, and these metals are likely controlled by similar processes as in the other sampling points, such as complexation and or adsorption (75).

## 4 Conclusion

Monitoring of water quality in five drinking water sources with different recharge mechanisms in the Caledonian nappes in Norway over the course of 17 months showed that apart from ^222^Rn, ID, and pH, most measured parameters were well within accepted norm for drinking water quality according to Norwegian drinking water regulations and European directives. However, observed activity concentrations of ^222^Rn ranged from <DL to 1,225 Bq/L, and was above the recommended water quality limit in one drilled granite well throughout the monitoring period. Among the other measured NOR, activity concentrations of ^210^Po up to 312 mBq/L were observed in the granite well. Consequently, the highest estimated possible ID was 0.31 mSv/y, calculated for the granite well water in a conservative exposure scenario for human intake. Water from the other groundwater sources and the stream showed lower concentrations of ^222^Rn and NOR. Thus, differences between sampling points were likely caused by a combination of geology, design of the source, and recharge mechanisms.

During the measurement period, which covered for all seasons, the six sampling points displayed different degrees of variation, in terms of both ^222^Rn, other NOR content and other water-parameters. Positive correlation between waterborne ^222^Rn and EC and major ions over time indicates that both are likely governed by maturity of groundwater and mixing, as was observed in the unconsolidated well. A positive correlation between ^222^Rn, REEs, and precipitation was observed in the granite well, and to some degree in the spring source. This is partly explained by transport along subterranean flow paths, although the recharge systems are likely more complex when compared to the unconsolidated well.

It is well known that airborne ^222^Rn is one of the main contributors to radioactive dose to the public, which makes mapping of ^222^Rn prone areas a necessary step towards reducing exposure. In terms of waterborne ^222^Rn, there exists a knowledge gap on both the effects of ingestion and its role in increased airborne activity. High risk areas can be identified with the help of geological maps alongside analyses of general water parameters and stable elements. Still, as this study shows there are challenges in prediction of waterborne ^222^Rn caused by differences in source type and seasonal variation in the different source types. Thus, highlighting the importance of direct measurements for monitoring purposes.

## Data Availability

The raw data supporting the conclusions of this article will be made available by the authors, without undue reservation.
